# Quantitative Analysis of PMLA Nanoconjugate Components after Backbone Cleavage

**DOI:** 10.3390/ijms16048607

**Published:** 2015-04-16

**Authors:** Hui Ding, Rameshwar Patil, Jose Portilla-Arias, Keith L. Black, Julia Y. Ljubimova, Eggehard Holler

**Affiliations:** Department of Neurosurgery, Cedars-Sinai Medical Center, 110 N. 127 S. San Vincente, Advanced Health Science Pavilion A8220, Los Angeles, CA 90048, USA; E-Mails: rameshwar.patil@cshs.org (R.P.); jose.portilla@cshs.org (J.P.-A.); keith.black@cshs.org (K.L.B.); julia.ljubimova@cshs.org (J.Y.L.); eggehard.holler@cshs.org (E.H.)

**Keywords:** polymalic acid, selective cleavage, PMLA nanoconjugate, quantitative analysis

## Abstract

Multifunctional polymer nanoconjugates containing multiple components show great promise in cancer therapy, but in most cases complete analysis of each component is difficult. Polymalic acid (PMLA) based nanoconjugates have demonstrated successful brain and breast cancer treatment. They consist of multiple components including targeting antibodies, Morpholino antisense oligonucleotides (AONs), and endosome escape moieties. The component analysis of PMLA nanoconjugates is extremely difficult using conventional spectrometry and HPLC method. Taking advantage of the nature of polyester of PMLA, which can be cleaved by ammonium hydroxide, we describe a method to analyze the content of antibody and AON within nanoconjugates simultaneously using SEC-HPLC by selectively cleaving the PMLA backbone. The selected cleavage conditions only degrade PMLA without affecting the integrity and biological activity of the antibody. Although the amount of antibody could also be determined using the bicinchoninic acid (BCA) method, our selective cleavage method gives more reliable results and is more powerful. Our approach provides a new direction for the component analysis of polymer nanoconjugates and nanoparticles.

## 1. Introduction

Polymer-nanoconjugates have great potential for drug delivery especially for the treatment of cancer [1,2]. Full-fledged single molecule versions of nanodrugs are designed to overcome multiple biological barriers *in vivo* by incorporation of multiple functional components [3,4]. Simple versions are chemically conjugated at least with a single targeting molecule and one drug molecule. Sophisticated versions may contain several such molecules including components functioning in endosomal escape and in protection against cleavage. Because of their increased complexity, quantitative chemical and functional characterization of constituents and the comparison with their free nonconjugated forms has become difficult due to the absence of appropriate methods. As a result, qualification for clinical applications has been greatly limited. Exhaustive analysis of components therefore is challenging and necessary.

Nowadays, nanoconjugates and nanoparticles are analyzed for size and Zeta-potential using light scattering techniques [5,6], and for morphology using transmission electron microscopy (TEM) [7]. Quantitative chemical and functional analysis of their components, however, affords multifaceted analysis, such as optical spectroscopy including labeling and reporter assays, nuclear resonance methods or biochemical/biological assays that account for the chemical/functional environment of the group of interest. Delicate groups such as proteins (antibodies) or peptides could suffer partial or complete loss of their activity due to side reactions during conjugation or platform induced shielding and intramolecular aggregation. Noncovalent structure based nanomaterials, such as liposome and micelle, and their loaded drugs are usually analyzed by dissolving the nanomaterials in organic solvent such as DMSO followed by assaying free drug and carrier components often using reversed phase HPLC [8]. In these cases, if a drug has characteristic UV maximum absorbance wavelength such as for doxorubicin, its amount can be estimated by UV absorbance [9]. In other cases, NMR has been used for estimation of covalently bound molecules [10]. But analysis is often limited by the lack of resolution due to molecular complexity. Thus, specific techniques have to be developed.

Polymalic acid (PMLA), a highly biocompatible and modifiable natural biopolymer, is an excellent nanoplatform for biocompatible drug delivery. The polyester is spontaneously and enzymatically degraded into L-malic acid which is ubiquitously incorporated into cell metabolic pathways and eventually metabolized to CO_2_ and H_2_O [11,12]. PMLA nanoconjugates showed no blood hematologic and immunologic toxicity after multiple intravenous administrations [13]. Due to its easy substitution at the pendant carboxylates, multifunctional PMLA conjugates have been prepared for the treatment of brain and breast cancer [14,15]. PMLA has been derivatized with various functional groups such as antibodies for tumor targeting [15,16,17] and crossing of the blood brain barrier (BBB) [14]. Morpholino antisense oligonucleotides (AONs) for inhibiting the synthesis of molecular tumor markers such as HER2, EGFR, laminin-411 [14,15], chemotherapeutic drugs [9,18], and amino acids active in endosome escape for cytoplasmic delivery [5,19]. PMLA-based nanoconjugates can be easily designed and synthesized for treatment of various cancers or malignancies and have been considered as very promising personalized medicine. Each individual component of nanoconjugates plays important and irreplaceable functions in the process of anticancer treatment. These nanoconjugates have a covalent all-in-one structure, and the quantitative chemical and functional analysis of the intact nanoconjugate has proven difficult and unreliable, especially when components have either poor UV resolution or spectral overlap. One such problem is the postsynthetic quantification of antibodies in a full nanoconjugate. The frequently used bicinchoninic acid (BCA) based protein assay applied to the intact nanoconjugate [20] could be inaccurate because of an inappropriate choice of protein standards and also because of an unknown contribution by other components. In this work, we report an unconventional approach including first the mild polyester backbone cleavage in aqueous solution of ammonia and then quantitative analysis of antibody and AON separated by SEC-HPLC with reference to free antibody and AON. We have developed this assay for the nanoconjugate P/mPEG(5%)/LLL(40%)/Herceptin(0.2%)/AON_HER2_(2%) (P is denoted as the PMLA backbone; mPEG as methoxypolyethylene glycol; LLL as trileucine; % refers to feed composition as the fraction of total malic acid units in polymer), which is an effective growth inhibitor in mouse models of human Her2/*neu* positive breast tumor [15]. The backbone cleavage by ammonia (ammonolysis) is selective for the polyester platform indicated by the full retention of Herceptin activity to bind HER2.

## 2. Results and Discussion

### 2.1. Selective Cleavage of PMLA

The synthesized nanoconjugate P/mPEG/LLL/Herceptin/AON_HER2_ has the potential for the treatment of Her2/*neu* positive breast cancer [15]. The purity of synthesis of the nanoconjugate was monitored with SEC-HPLC in each step. The size and zeta-potential of the nanoconjugate was 24 nm and −9 mV. The goal of this study was to develop a method for quantitation of antibody and AON in the nanoconjugate by applying a mild cleavage condition for selective degradation of the nanoconjugate platform.

**Figure 1:**
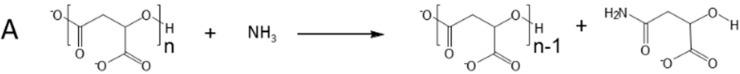

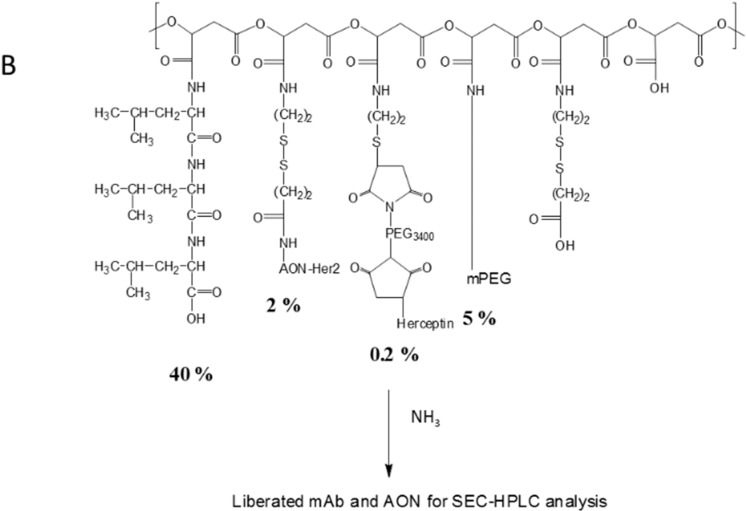
Cleavage of PMLA (**A**) and multifunctional nanoconjugate (**B**) by mild ammonolysis in aqueous solution.

**Figure 2:**
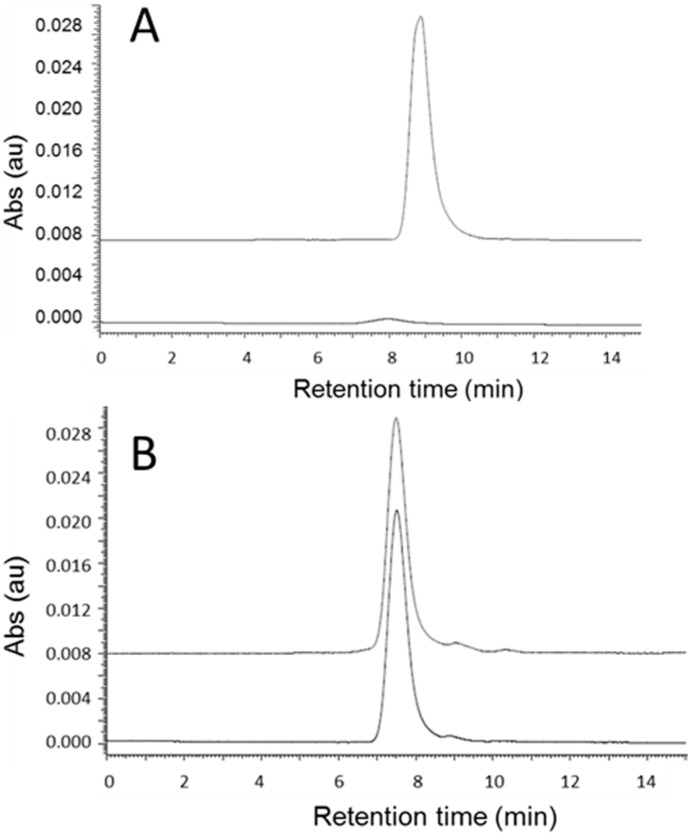

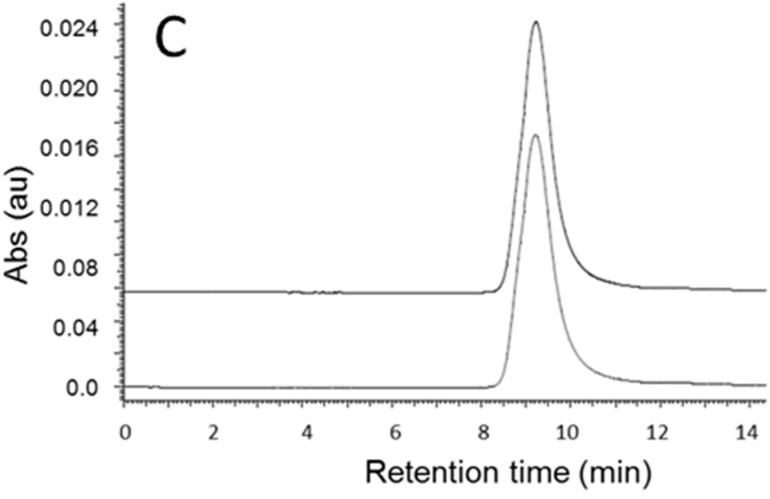
SEC-HPLC analysis of (**A**) PMLA before (lower trace) and after (upper trace) standard ammonolytic treatment at 220 nm wavelength; (**B**) Herceptin before (lower trace) and after (upper trace) standard ammonolytic treatment at 280 nm wavelength; and (**C**) AON before (lower trace) and after (upper trace) standard ammonolytic treatment at 260 nm wavelength.

Poly(β-l-malic acid) (PMLA) can be cleaved by aqueous ammonia to convert backbone ester into oligomers and ultimately monomers involving nucleophilic attack of the backbone ester carbonyl group (Figure 1A) [21,22]. In a first approach we found that a mixture of 3% of sodium hydroxide and 30% of ammonium hydroxide cleaved PMLA quantitatively within 2 h at room temperature. However under the same conditions Herceptin was also degraded. In a second approach, controlled degradation was achieved under standard cleavage conditions, *i.e.*, by mixing PMLA in an equal volume of phosphate buffer (pH 6.3, final concentration 50 mM) with ammonium hydroxide (final concentration 0.5%), 3 mg/mL dithiothreitol (DTT) and 24 h incubation at room temperature. The resulting pH of 9.6 is tolerable for most antibodies. PMLA cleavage was accompanied by a shift in SEC-HPLC elution peak from before 7.8 min (Figure 2A lower) to 9.2 min (Figure 2A upper) at the end of the experiment. The shifted position corresponded with the prolonged retention time for malic acid. The intensity was enhanced by a factor of more than 20-fold, typical for the cleavage of polymalic acid [21,22]. In contrast, antibody with and without treatment (24 h, 50 mM phosphate buffer, 3 mg/mL DTT, 0.5% ammonium hydroxide, final pH 9.6) exhibited conserved UV_280_ absorbance, SEC-HPLC retention and polydispersity PD = 1.07 (Figure 2B lower and upper) suggesting antibody integrity.

Next, we examined whether Morpholino AON_HER2_ alone was resistant to the cleavage condition. The oligonucleotide 2 mg/mL was incubated in the established cleavage mixture (chapter 2.3.) of 50 mM (pH 6.3) phosphate buffer, 0.5% ammonia, 3 mg/mL DTT for 24 h at room temperature. Elution profiles from reversed phase HPLC (260 nm) before and after treatment were found indistinguishable, and the oligonucleotide was considered not degraded. In addition, its SEC-HPLC profile was identical before and after ammonolytic treatment (Figure 2C).

### 2.2. Cleavage Kinetics for Free PMLA

**Figure 3:**
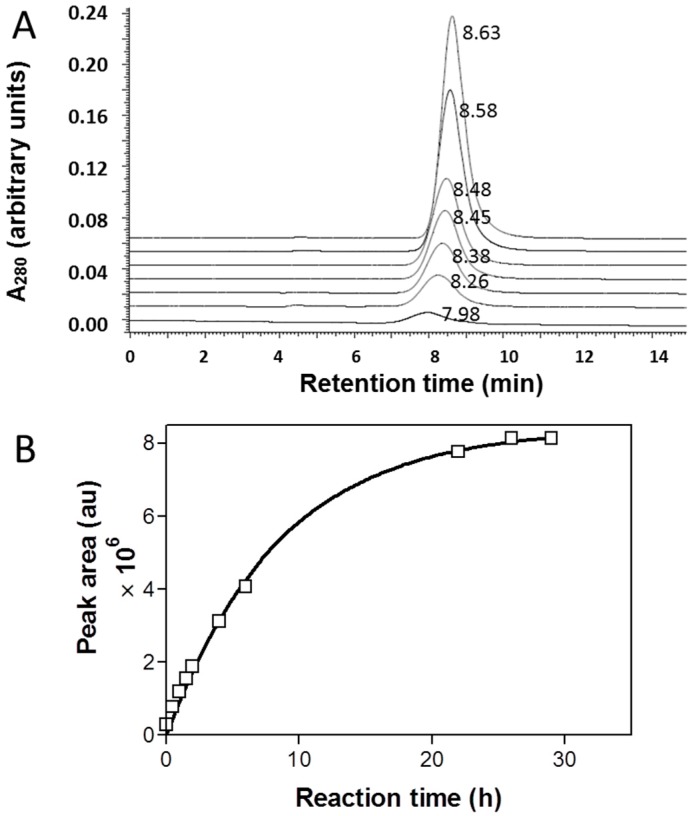
Kinetics of PMLA cleavage under condition of standard ammonolysis. At various times (bottom 0, 0.5, 1, 1.5, 2, 4, and 6 h top), samples were analyzed by SEC-HPLC and retention time is indicated next to each peak (**A**); The areas under the peaks were plotted against incubation time (**B**).

During the treatment of PMLA under standard conditions with 0.5% aqueous ammonium hydroxide, the backbone was gradually degraded into fragments (Figure 3). To find out the requirement for reaction completion, the time course was followed by SEC-HPLC at various time points. The cleavage was indicated by a shift of the elution peak to the right indicating the fragmentation of the polymer (Figure 3A). The absorbance increase is consistent with the cleavage of the intra-polymer ester bonds typically observed for amide and carboxylate formation through nucleophilic carbonyl attack by NH_3_ or OH^−^ present in the reaction mixture. To obtain the desired kinetic information, peak areas were plotted against time and the data fit exponentially using Graphpad Prism 3.0 (GraphPad-Prism Software Inc., La Jolla, CA, USA). The estimated cleavage half-life was 5.9 h (Figure 3B). As indicated by the time dependence, the degradation of PMLA was close to completion after 24 h and this time was used as standard cleavage condition.

### 2.3. Cleavage of P/mPEG(5%)/LLL(40%)/Herceptin(0.2%)/AON_HER2_(2%)

**Figure 4:**
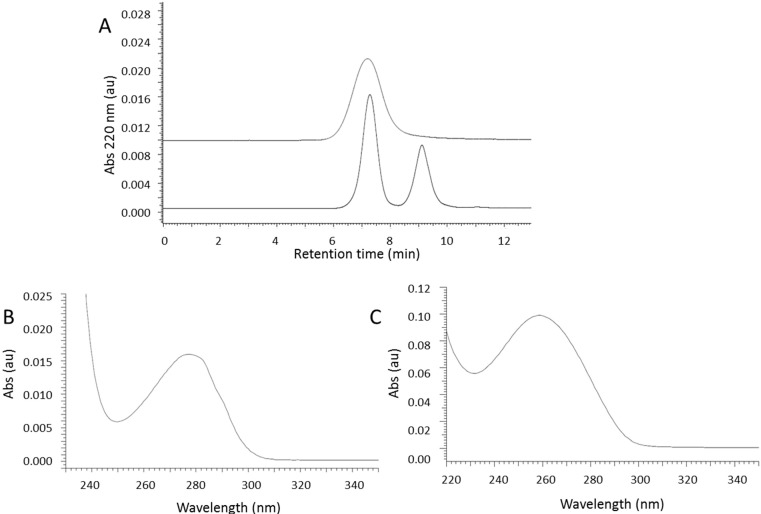
Selective cleavage of PMLA nanoconjugate followed by SEC-HPLC. (**A**) SEC-HPLC (upper trace) at 220 nm wavelength showing the nanoconjugate before cleavage indicated by a single broad peak. After cleavage, two peaks emerged (lower trace); The first peak shows the absorbance profile of Herceptin and a maximum at 280 nm wavelength (**B**); The second peak shows the absorbance spectrum for AON and a maximum at 260 nm (**C**).

Mechanistically, ammonolytic ester cleavage engages nucleophilic substitution of the ester bond resulting in PMLA cleavage and formation of malamide and in parallel ester hydrolysis due to the slightly alkaline conditions of the reaction mixture (pH 9.6). SEC-HPLC analysis of intact nanoconjugate showed a single peak at 7.2 min measured at 220 nm wavelength (Figure 4A upper trace). This was the superposition of peaks for Herceptin measured at 280 nm wavelength and AON_HER2_ measured at 260 nm wavelength, when both were conjugated to PMLA. Cleavage of nanoconjugate with 0.5% ammonium hydroxide alone resulted in two major SEC-HPLC peaks at 7.2 and 9.2 min, and a third minor peak at 8 min. Peaks at 8.0 and 9.2 min had the characteristic UV absorbance spectrum of AON. We have considered the possibility of an AON fragment containing incompletely digested large fragments of the polymer platform. To remove the fragment, we added 3 mg/mL DTT that would cleave the polymer fragment and result in the elimination of the peak at 8.0 min. This presence of DTT is included in the standard cleavage condition (see Experimental Section).

After cleavage under standard condition, fragments eluted in two well separated SEC-HPLC peaks recorded at 220 nm wavelength in Figure 4A (lower), one at 7.5 min and the other at 9.2 min. The 7.2 min peak resembled the elution of free Herceptin by maximal absorbance at 280 nm wavelength (Figure 4B), and the other at 9.2 min the elution of AON_HER2_ by maximal absorbance at 260 nm (Figure 4C). The profile assigned to Herceptin (Figure 4A lower trace) was much sharper (polydispersity PD = 1.11 by SEC-HPLC) than the elution peak for the intact nanoconjugate (Figure 4A upper trace, PD = 2.3).

### 2.4. Quantitation of Antibody (Herceptin) and AON_HER2_

Ultra violet light absorbance at 260 and 280 nm wavelength is negligible for PMLA and fragments containing malic acid, trileucine and mPEG in comparison with the absorbance by Herceptin or AON_HER2_. We therefore considered quantifying Herceptin and AON on the basis of absorbance units under their respective SEC-HPLC elution peaks at 7.2 and 9.2 min. Standard curves (correlation coefficient > 0.99) were established by measurement of areas under the corresponding SEC-HPLC elution peaks of free Herceptin and AON respectively. Amounts of Herceptin and AON obtained by this method were compared with the gravimetrically measured amounts used in the synthesis of the nanoconjugate and are shown together with the calculated recovery yields in Table 1. The amounts of Herceptin and AON_HER2_ thus evaluated were 5.05 ± 0.10 mg and 1.45 ± 0.05 mg that compared with 5.0 ± 0.1 mg Herceptin and 10 ± 0.1 mg AON fed into synthesis translating to 101% recovery for Herceptin and 14.5% for AON_HER2_. A similar value of 17% for the recovery for AON was confirmed after reductive cleavage of the nanoconjugate by DTT and quantitation of the released AON by reversed phase-HPLC using an established method [23]. The recovery for AON reflected the yield of conjugation with PMLA through disulfide bond that is typically low [24]. In order to favor a high loading of AON, it has been found practically to use a large excess of 3-[2-pyridyldithio]-propionate modified AON (PDP-AON) over thiol groups in the nanoconjugate.

**Table 1:** Quantitative analysis of nanoconjugate components after ammonolysis.

	Herceptin	AON
Sample, conc (mg/mL)	1.01 ± 0.03 ^a^	0.29 ± 0.01 ^a^ 0.34 ± 0.01 ^d^
Synthesis, feed (mg)	5.00 ^g^	10.00 ^g^
Content, measured (mg)	5.05 ± 0.15 ^b^	1.45 ± 0.05 ^b^ 1.7 ± 0.05 ^e^
Recovery (%)	101 ^c^	14.5 ^c^ 17.0 ^f^

^a^ Concentration; ^b^ total content; and ^c^ recovery determined by ammonolysis and SEC-HPLC analysis; ^d^ AON concentration; ^e^ total content; and ^f^ recovery determined by reductive cleavage and reverse phase HPLC analysis; ^g^ Feed for synthesis measured by gravimetry.

### 2.5. Quantitation of Herceptin in the Nanoconjugate Using the BCA Method

**Figure 5:**
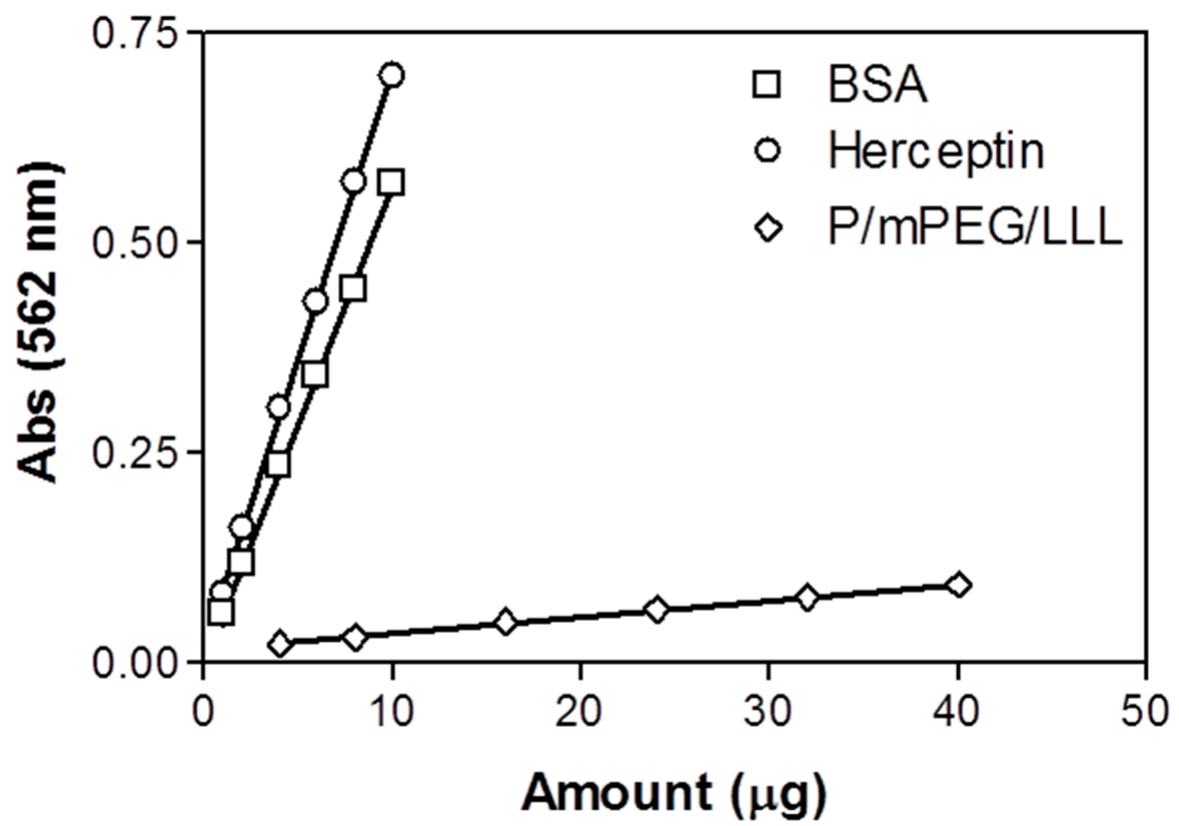
Standard curves for Herceptin, BSA, and P/mPEG/LLL obtained using BCA method. Data are triplicate with standard deviation.

The development of the new method for protein quantification of PMLA nanoconjugates was driven to circumvent unaccounted variations due to the presence of attached amino acid derivatives such as trileucine or leucine ethylester and due to the choice of protein standard such as BSA when applying a typical colorimetric protein method. When using the BCA assay, variations were observed in our example originating from PMLA-attached trileucine and from using BSA are illustrated in Figure 5. The contribution by trileucine seems to be marginal, whereas selection of BSA as standard significantly increased the estimated value of the protein that would have been correctly obtained with Herceptin as standard. In fact when we used BCA method and Herceptin as standard we measured 1.05 ± 0.04 mg/mL as the amount of Herceptin attached to the nanoconjugate compared with 1.01 ± 0.03 mg/mL by our ammonolytic cleavage/HPLC method. The result confirmed the notion that in the BCA assay the protein referenced should be identical with the protein in the sample.

### 2.6. Characterization by ELISA of Herceptin after Nanoconjugate Ammonolysis

**Figure 6:**
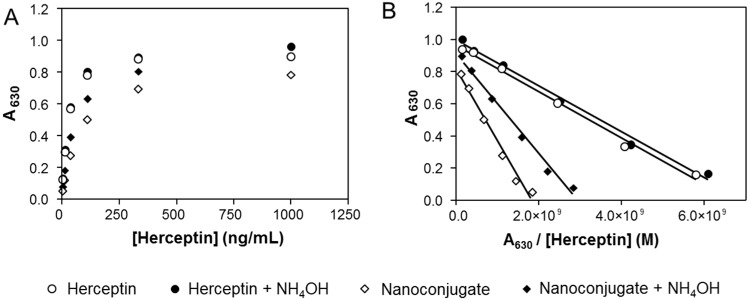
ELISA analysis of Herceptin and nanoconjugate before and after standard ammonolytic treatment. (**A**) Readout from ELISA; (**B**) Replot of ELISA according to Hofstee [24] with *A*_630_/[C] as *x*-axis and *A*_630_ as *y*-axis, where *A*_630_ is the absorbance reading at 630 nm wavelength, and [C] is the concentration of antibody in molar concentration. Data are triplicates with the magnitude of standard deviations not exceeding the dimension of the graphical symbols. Symbols refer to O, Herceptin; ●, Herceptin after treatment; ◆, Polymer nanoconjugate after treatment; ◇, PMLA nanoconjugate without treatment.

Herceptin recovered after nanoconjugate ammonolysis exhibited properties comparable with that of the free untreated antibody. To learn whether recognition and HER2-binding affinity were conserved, an ELISA using HER2 as antigen was performed. The samples were both Herceptin and nanoconjugate with/without ammonolytic treatment. In Figure 6A it is evident that in all samples, the concentration dependencies reflected similar binding affinities (Figure 6A). For quantitative analysis, the data were re-plotted according to Hofstee [25] with *A*_630_/[C] as *x*-axis and A_630_ as *y*-axis, where *A*_630_ is the absorbance reading at 630 nm, indicating the amount of formed HER2-Herceptin (nanoconjugate) complex, and [C] is the molar concentration of the antibody (Figure 6B). The experimental data was fit to the linear equation *A*_630_ = *A*_630_(tot) − *K*_d_ × A_630_/[C], where *A*_630_(tot) (by extrapolation) is interpreted as maximum absorbance by ligand saturation and *K*_d_ (from the slope) the dissociation constant of the receptor-ligand complex. In each case, the perfect linear fit confirmed the assumption of HER2-Herceptin/nanoconjugate complexes. The dissociation constant for Herceptin with ammonolysis was 1.44 × 10^−10^ M, identical to the one without treatment (*K*_d_ = 1.43 × 10^−10^ M). Binding affinity of Herceptin in the nanoconjugate was lower than for free Herceptin indicated by the higher value of *K*_d_ (4.5 × 10^−10^ M). This was the result of shielding by the polymer after conjugation. After ammonolysis of nanoconjugate, the *K*_d_ value (3.1 × 10^−10^ M) was much smaller than that of the intact nanoconjutate suggesting reconstitution of Herceptin binding affinity after its liberation from the backbone. The value of *A*_630_(tot) for nanoconjugate after treatment equals the one for free Herceptin and is 20% higher than the value for intact nanoconjugate (Figure 6B), suggesting the avidity of Herceptin was fully recovered after backbone cleavage. The ELISA results therefore confirmed the selective cleavage of PMLA without major physical and biological damage to the protein and the application of SEC-HPLC after ammonolysis is a practicable method to analyze Herceptin and most likely other antibodies/proteins after their covalent incorporation into PMLA nanoconjugates.

## 3. Experimental Section

### 3.1. Materials

Poly(β-l-malic acid) (PMLA) (50 kDa; on average 431 malic acid residues, polydispersity 1.3) was obtained from culture broth of the myxomycete *Physarum polycephalum* as described [11,12,17]. Trileucine H-Leu-Leu-Leu-OH (LLL) was purchased from Bachem Americas Inc (Torrance, CA, USA). Methoxypolyethylene glycol (mPEG) was obtained from Laysan Bio (Arab, AL, USA) and Pierce BCA protein assay kit from Thermo Scientific (Rockford, IL, USA). Morpholino antisense oligo 5'-CATGGTGCTCACTGCGGCTCCGGC-3' (AON_HER2_) was custom made by Gene Tools (Philomath, OR, USA). *N*-Succinimidyl 3-[2-pyridyldithio]-propionate (SPDP), glycine buffer (catalog No. G5418), dithiothreitol (DTT) were purchased from Sigma Aldrich. Herceptin (Trastuzumab) was purchased from the pharmacy at Cedars-Sinai Medical Center. Extracellular domain (ECD) of the HER2/neu receptor was a gift from Manuel Penichet (University of California at Los Angeles, CA, USA).

### 3.2. Synthesis of P/mPEG(5%)/LLL(40%/Herceptin(0.2%)/AON_HER2_(2%)

The PMLA nanoconjugate P/mPEG(5%)/LLL(40%/Herceptin(0.2%)/AON_HER2_(2%) was used for developing the selective cleavage condition. It was synthesized by conjugating preconjugate P/mPEG(5%)/LLL(40%)/MEA(10%) with Herceptin-PEG_3400_-Mal and PDP-AON following the previously described method [14,23]. The nanoconjugate contains five key components (Figure 1B): PMLA as the backbone; 2% Morpholino antisense oligonucleotide (AON_HER2_) to inhibit HER2/neu protein synthesis; 0.2% Herceptin, 40% *tri*(l-leucine) (LLL) for cytoplasmic delivery of AON_HER2_, and 5% mPEG_5000_ to increase serum stability. Herceptin, 5 mg, was conjugated to 8.6 mg preconjugate via thioether formation of Mal-PEG_3400_-Mal linker, followed by conjugation with 10 mg PDP-AON_HER2_. The synthesized nanoconjugate was purified by size exclusion chromatography (GE Healthcare, Uppsala, Sweden) on Sephadex G-75 column with PBS as eluent at room temperature. Nanoconjugate collected in 5 mL PBS was used as the stock solution for developing the quantitative assay. Hydrodynamic diameter and zeta potential were measured by dynamic light scattering in PBS using a Zetasizer Nano-ZS90 (Malvern Instruments, Malvern UK). The solutions of nanoconjugate were prepared in PBS at a concentration of 1 mg/mL, filtered through a 0.2 μm pore membrane. All the copolymer solutions were prepared immediately before analysis at 25 °C.

### 3.3. Selective Cleavage of the Nanoconjugate Backbone by Ammonium Hydroxide

The standard cleavage reaction at room temperature was carried out as follows: stock solution of nanoconjugate P/mPEG/LLL/Herceptin/AON_HER2_ in PBS 150 µL was mixed with 150 µL of phosphate buffer final concentration 50 mM pH 6.3. To this solution 3 mg/mL DTT was added and the reaction tube agitated for 30 min. To the mixture, 5 µL of ammonium hydroxide (30%) at final concentration of 0.5% was added. The extent of cleavage was assayed by SEC-HPLC with UV detector at 220, 260, and 280 nm wavelength using Hitachi Elite LaChrom HPLC system at 25 °C, equipped with pump L-2130, diode array detector L-2455, PolySep-GFC-P 4000 column (300 mm × 7.8 mm) and PBS as mobile phase (flow rate 1 mL/min). After 24 h at room temperature the reaction mixture was analyzed by SEC-HPLC to assess the concentrations of antibody and AON.

### 3.4. Quantitation of Antibody by SEC-HPLC in the Reaction Mixture after Cleavage

For quantitation, 20 µL of the ammonolytic cleavage mixture was analysed on Hitachi Elite LaChrom HPLC system at 25 °C, equipped with pump L-2130, diode array detector L-2455, PolySep-GFC-P 4000 column (300 mm × 7.8 mm) and PBS as mobile phase (flow rate 1 mL/min), UV detection at 280 nm and software D-2000 Elite. The content of antibody was quantified by measuring the area under the SEC-HPLC elution peak referenced to various concentrations of commercial Herceptin as standards. The method was highly reproducible in support of the excellent HPLC resolution.

### 3.5. Herceptin Quantitation Using the BCA Protein Assay Prior to Cleavage

The amount of Herceptin in nanoconjugate prior to cleavage was measured by the colorimetric bicinchoninic acid (BCA) method [26] using a commercial kit from Pierce based on colorimetric reactions of peptide bonds and side chains of cysteine, cystine, tryptophan and tyrosine [27]. Different amount of Herceptin, BSA, and P/mPEG(5%)/LLL(40%) 20 µL in triplicate was added to wells of a plate followed by the addition of BCA working reagent 200 µL according to manufacturer’s protocol. The mixture was incubated at 37 °C for 30 min and the absorption of samples was read at wavelength 562 nm in a SPECTRAmax M2 (Molecular Devices, Sunnyvale, CA, USA) equipped with SoftMax Pro 5.4. Standard curves in triplicate for Herceptin, BSA, and P/mPEG/LLL were established usingsoftware Graphpad Prism 3.0.

### 3.6. ELISA for Free and Conjugated Herceptin

The antigen binding activity of free and conjugated Herceptin, nanoconjugate P/mPEG/LLL/Herceptin/AON_HER2_, before and after treatment with ammonium hydroxide was analyzed using protein detector ELISA kit (KPL, Maryland, MD, USA). The Nunc MaxiSorp plate was coated with ECD-HER2 receptor, 100 ng, in 50 µL/well coating buffer by incubation for 2 h at room temperature. After blocking with 4% milk for 40 min, the plate was rinsed with washing buffer. Four independent sets of samples were plated after serial dilution and incubated for 1h at room temperature. The plate was washed repeatedly and incubated with secondary antibody, anti-goat human/HRP conjugate (1:2000), for 1 h, washed again, and incubated with peroxidase substrates for 30 min. Absorbance indicating the formation of HER2-Herceptin complex was read at wavelength 630 nm in a SPECTRAmax M2 (Molecular Devices) equipped with SoftMax Pro 5.4. Absorbance as function of various Herceptin concentrations were plotted according to Eadie-Hofstee [25]. From the slope of the linear best fit, the dissociation constant of the Herceptin-HER2 antibody-receptor complex was calculated.

## 4. Conclusions

Component analysis of polymer nanoconjugtes is usually circumstantial and difficult depending on its chemistry and complexity. PMLA is an optimal choice of platform to achieve both high functionality and post-synthetical chemical validation. An outstanding advantage is selective cleavage of the polymer backbone in the presence of dilute ammonia. At selected conditions, only the backbone is degraded without affecting the integrity of antibody and Morpholino antisense oligonucleotide which are separated and quantified in a single step SEC-HPLC analysis. Auxiliary methods are not required and it is sufficient to follow UV absorbance of antibody and antisense oligonucleotide. Ambiguities inferred by inappropriate standards when using protein assays can be avoided rendering quantitation highly reliable. Our analytical approach provides a new direction for functional component analysis in favor of polymalic acid based biodegradable, non-toxic and non-immunogenic nanoconjugates/particles due to its unique backbone chemistry.
